# P-1774. Clinical presentation of Cutaneous Leishmaniasis (CL) in returning travelers

**DOI:** 10.1093/ofid/ofaf695.1944

**Published:** 2026-01-11

**Authors:** Olivia Man, Mark Enzler, Bobbi S Pritt, Abinash Virk, Omar M Abu Saleh

**Affiliations:** Mayo Clinic, Rochester, Minnesota; Mayo Clinic College of Medicine, Rochester MN, Rochester, Minnesota; Mayo Clinic, Rochester, Minnesota; Mayo Clinic, Rochester, Minnesota; Mayo Clinic, Rochester, Minnesota

## Abstract

**Background:**

Leishmaniasis is a rare protozoan parasitic disease transmitted through the bite of infected Phlebotomus and Lutzomyia sandflies. Clinical presentation is variable due to numerous species and host immunity differences. We present our clinical experiences with CL to guide timely diagnosis and treatment in returning travelers.

**Figure:**
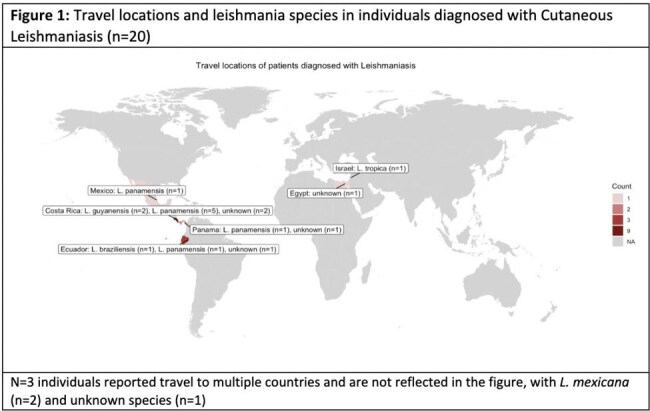

**Figure:**
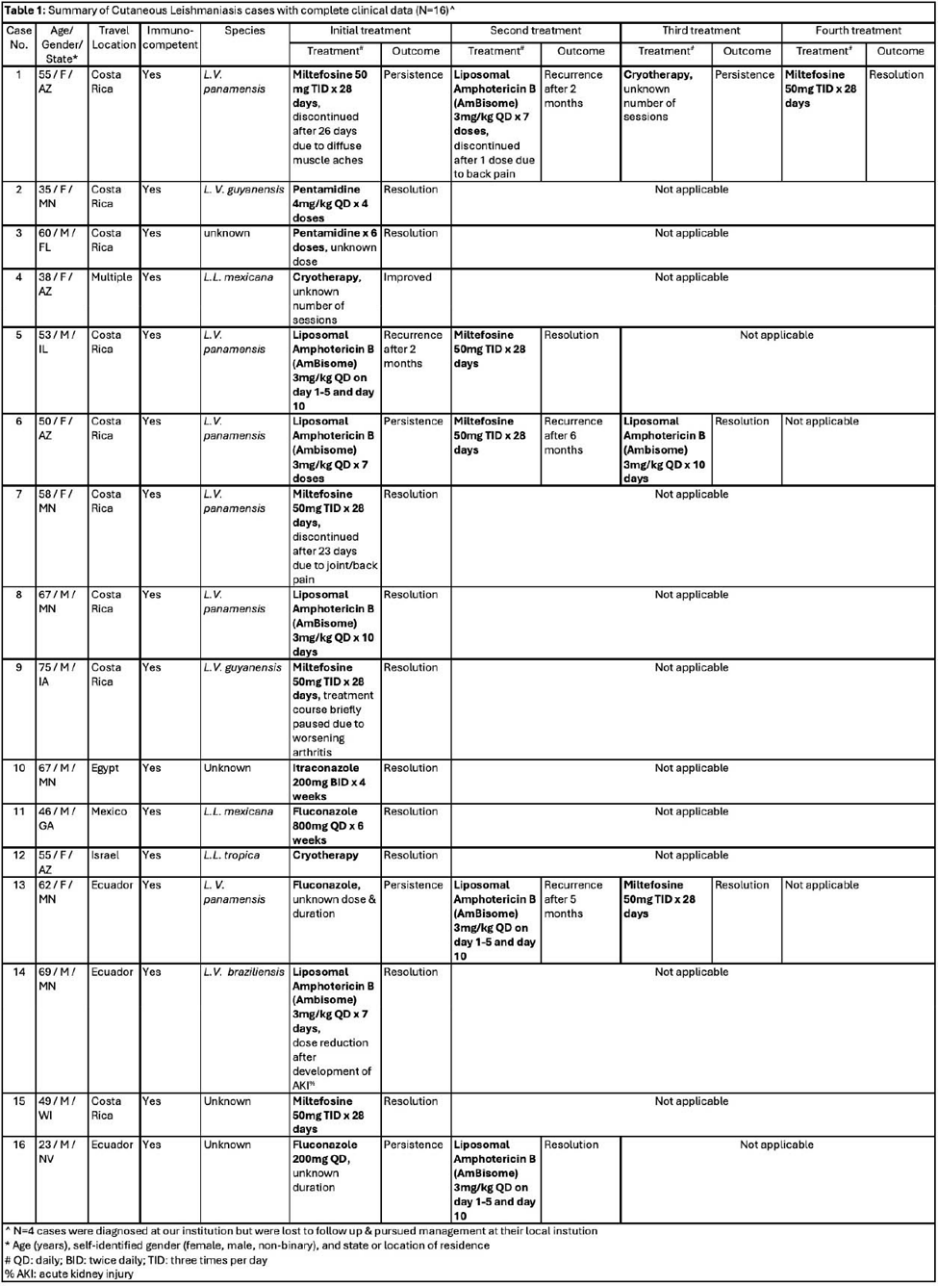

**Methods:**

We retrospectively analyzed a cohort of adult patients 18+ years old who were diagnosed with CL from 2005 and 2025. We describe the demographic, clinical, and diagnostic features of CL.

**Figure:**
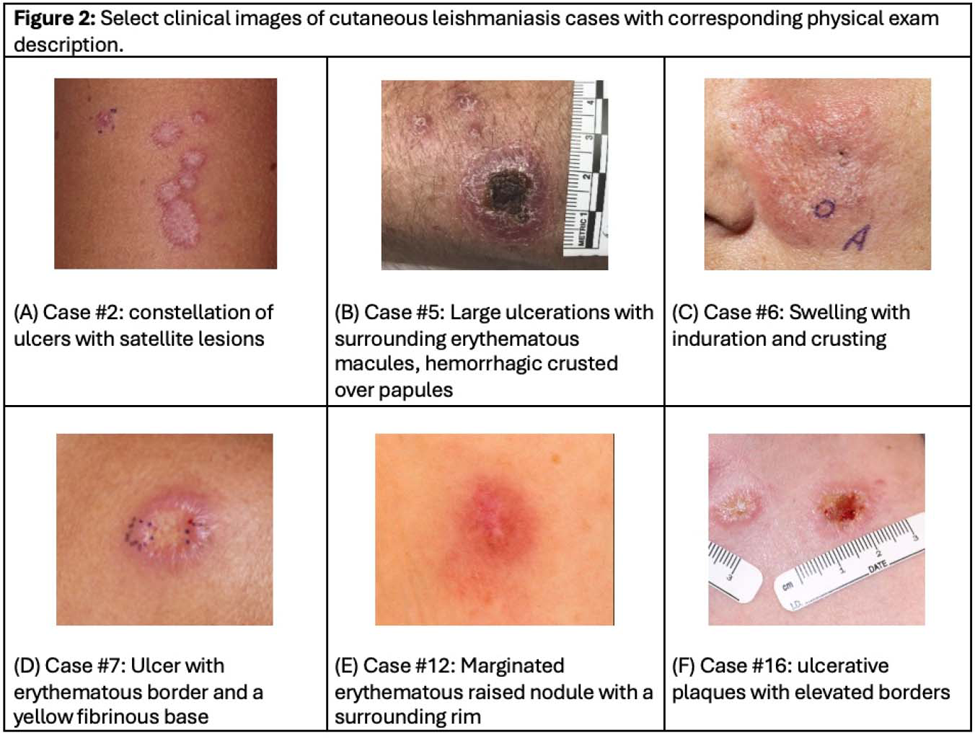

**Results:**

Biopsy-confirmed CL was diagnosed in 20 returning travelers (Table 1) from various endemic regions, most commonly Costa Rica (Fig. 1). The median time from travel to symptom onset was 36.5 days (SD 102), with an additional 75.5 days (SD 87) prior to biopsy diagnosis. All patients were evaluated by dermatology and 70% were evaluated by infectious disease (n=14). On initial presentation, lesions were described as ulcerated (50%, n=10), nodular (30%, n=6), painful (20%, n=4), and necrotic (10%, n=2; Fig. 2). Most lesions were located on the upper extremity (55%, n=11), followed by the face (35%, n=7), lower extremity (30%, n=6), and trunk (15%, n=3). 15% of individuals (n=3) had constitutional symptoms. No patients had definitive mucosal involvement, however, 10% (n=2) endorsed lip edema. No patients had visceral involvement. Species level identification occurred in 14 cases (70%), of which the most common was L.V. panamensis (50%), L.L. mexicana (21%), L. V. guyanensis (14%), L. L. tropica (7%), and L. V. braziliensis (7%). Initial treatments included azoles, liposomal amphotericin B (AmBisome), and miltefosine (each 20%); cryotherapy and parenteral pentamidine (each 10%); with compounded topical paromomycin added in 15% of cases. 35% of individuals (n=7) failed initial treatment and required additional treatment courses due to persistent lesions (57%) or relapse following initial resolution (14%). 10% (n=2) required a third treatment course due to persistent/recurrent lesions.

**Conclusion:**

While CL in travelers has a variety of clinical manifestations, travel history, knowledge of key clinical features, and lesion patterns can aid in early diagnosis and treatment. CL is associated with early treatment failure rate for uncertain reasons.

**Disclosures:**

All Authors: No reported disclosures

